# Enhanced Donor Lung Viability During Prolonged *Ex Vivo* Lung Perfusion Using ECMO Technology

**DOI:** 10.3389/ti.2025.14284

**Published:** 2025-10-02

**Authors:** Linar Faizov, Yerik Zuparov, Aiym Turarova, Zhuldyz Nurmykhametova, Aidyn Kuanyshbek, Rymbay Kaliyev, Anatoly Sergienko, Timur Lesbekov, Yuriy Pya

**Affiliations:** ^1^ Department of OR with Mechanical Circulatory Support, National Research Cardiac Surgery Center, Astana, Kazakhstan; ^2^ Department of Bioengineering, National Research Cardiac Surgery Center, Astana, Kazakhstan

**Keywords:** normothermic machine perfusion, Cytosorb, *ex vivo* lung perfusion, ECMO technology, bronchodilation

## Abstract

The shortage of donor organs remains a significant obstacle to addressing the growing demand for lung transplantation. We have developed a mobile EVLP circuit utilizing extracorporeal membrane oxygenation (ECMO) technology. The goals of our study was to evaluate the safety of a cellular-based EVLP system and to assess the efficacy of continuous cytokine removal and the application of bronchodilators during prolonged EVLP. A total of 29 pigs, aged 6–18 months and weighing 93 ± 13.1 kg (mean ± SD), were used in the study. The lungs were randomly allocated into the following groups: Cytosorb group (n = 12), were perfused with an EVLP circuit containing a Cytosorb cartridge, and Control group (n = 12), where lungs were perfused with an EVLP circuit without a Cytosorb cartridge, Bronchodilator group (n = 5) consisted of lungs that were nebulized with a combined bronchodilator every 4 h after the initiation of ventilation and Control group (n = 5) inhaled the control solvent under the same settings. Our study demonstrates that whole blood based perfusate with continuous ultrafiltration for functioning lung in machine perfusion can be straightforward and efficient for at least 24 h. Additional cytokine removal had led to significant improvement of organ quality over 24 h of EVLP.

## Graphical Abstract

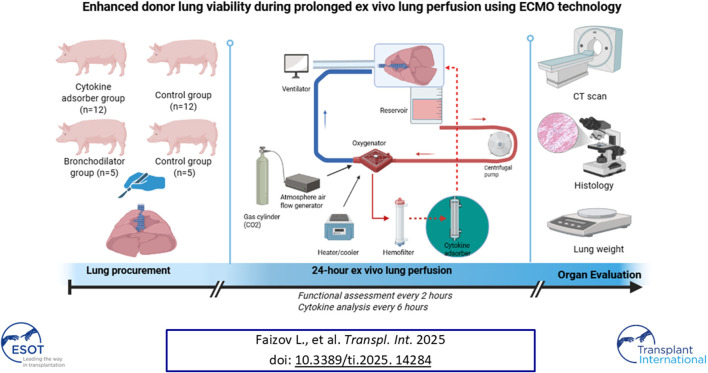

## Introduction

The shortage of donor organs remains a significant obstacle to addressing the growing demand for lung transplantation (LTx). Despite an annual increase in the number of lung transplants performed, approximately 18.8% of transplant candidates are removed from the waiting list each year due to the lack of suitable donor lungs [1]. *Ex vivo* lung perfusion (EVLP) technology, introduced globally by Steen et al. in the mid-2000s, has revolutionized the field by expanding the donor pool without compromising recipient outcomes [2, 3]. This innovative technique preserves lungs in a physiological state before transplantation, allowing for their evaluation, preservation, and reconditioning. Unlike cold static preservation, normothermic perfusion sustains the metabolic activity and viability of lung cells for extended periods. Various EVLP protocols are now in clinical use, employing different types of perfusates. Research suggests that cellular perfusates offer advantages over acellular perfusates, including reduced edema formation [4–6], lower levels of inflammatory cytokines [7], and decreased expression of genes associated with acute lung injury (ALI) [8, 9]. The TransMedics Organ Care System (OCS) has demonstrated effective use of donor blood for *ex vivo* lung preservation, reducing ischemic time, minimizing blood wastage, and enhancing the efficiency of organ retrieval and transplantation processes [10]. However, despite its significant benefits, the high costs associated with this technology and its consumables may limit its broader adoption [11, 12]. Primary graft dysfunction (PGD) is a major cause of transplant-related morbidity and mortality, primarily driven by ischemia-reperfusion injury (IRI), which triggers oxidative stress, inflammation, and alveolar damage [13–15].

To address these challenges, we developed a mobile EVLP circuit based on extracorporeal membrane oxygenation (ECMO) technology. The primary goal of this study was to evaluate the safety of a novel, easily transportable, cellular-based EVLP system. A secondary goal was to assess the efficacy of continuous cytokine removal and the application of bronchodilators during prolonged EVLP.

## Materials and Methods

### Animal Model

Experiments were conducted in accordance with the guidelines of the National Research Cardiac Surgery Center’s Animal Care and Use Committee (Protocol No 2022/01-121). A total of 29 pigs (*Sus scrofa* ssp. domesticus), aged 6–18 months and weighing 93 ± 13.1 kg (mean ± SD), were used in the study. Prior to surgery, food was withheld for each pig for 24 h and water for 12 h. Thiopental sodium (10–15 mg/kg), administered through an angiocatheter to induce an anesthesia. Animals were intubated and mechanical ventilation was initiated in volume control mode with a tidal volume of 8 mL/kg, peak inspiratory pressure (PIP) between 18–20 cmH2O, and positive end-expiratory pressure (PEEP) of 5 mmHg. Ventilation settings were adjusted to maintain normocapnia, ensuring that the partial pressure of carbon dioxide (PaCO2) remained within the target range of 35 mmHg–45 mmHg. Anesthesia was maintained using isoflurane, titrated to achieve a minimum alveolar concentration (MAC) of 0.5%–1.5%. Vital parameters, including heart rate, invasive blood pressure, central venous pressure, oxygen saturation, and end-tidal CO2, are monitored continuously.

### Lung Procurement

A median sternotomy was performed, followed by exsanguination via right atrial cannulation. Systemic anticoagulation was achieved with an intravenous bolus of 50,000 IU heparin sodium (Chempharm, Kazakhstan). Prior to lung procurement, a recruitment maneuver was applied. The porcine lungs were harvested with the trachea clamped at a peak inspiratory pressure (PIP) of 25 cmH2O. The pulmonary arteries were flushed in both anterograde and retrograde directions with 1,000 mL of Plasmalyte solution (Baxter Polska, Warszawa) to remove residual blood. After harvesting, a collapse test was performed to evaluate the biomechanical properties of the lungs. To ensure safe cannulation of the pulmonary artery to the EVLP circuit, a 3–4 cm graft was attached to elongate the vessel. Following this, the lungs were attached to the EVLP system and perfused for 24 h. Reperfusion of the *ex vivo* lungs began approximately ±50 min after aortic cross-clamping.

### *Ex Vivo* Lung Perfusion

The EVLP system was primed with blood from the donor pig, human Albumin 20% - 500 mL (Biopharma, Ukraine) and diluted to achieve a mean hematocrit of 10%–15% (2% standard error, hereafter SE), making a total volume of approximately 1L. The heat exchanger integrated in the oxygenator was used to keep the perfusate at normothermia and facilitated gas exchange using 0.1–0.2 L per minute (LPM) of atmosphere air flow with mixed CO2 30–50 mL/min. The system was then de-aired and circulation started at a low flow 0.5 L/min, than perfusion flow during 24 h was 0.8–1.0 L/min. Pharmacological support provided via controlled on demand infusion of glucose, insulin, sodium nitroprussid and sodium bicarbonate, methylprednisolone 250 mg (Pfizer, USA). Hemoglobin and glucose were checked every 30 min during EVLP. Similarly, the perfusate was maintained at a normal range of pH = 7.4 (SD 0.1).

Active zero balance ultrafiltration with Plasmalyte solution (Baxter Polska, Warszawa) as a plasma substitution solution was used to maintain the hematocrit and electrolytes in the target normal range.

The EVLP circuit utilized extracorporeal membrane oxygenation (ECMO) technology. The system included a chamber designed for the harvested lung, equipped with an integrated reservoir to facilitate passive drainage of pulmonary venous blood. Continuous perfusion was maintained using a centrifugal pump, with the perfusate flow rate set at 30% of the estimated cardiac output. To deoxygenate the perfusate, carbon dioxide was intermittently (30–50 mL/min) introduced into the oxygenator. The perfusate temperature was regulated at 37 °C using a heat exchanger (made in our institution) to optimize cellular metabolism during perfusion. A hemofilter (B.Braun A vitrum AG, Germany) was integrated into the circuit with a flow rate of 30 mL/min to allow continuous hemodialysis of the perfusate across all experimental groups. After mounting the lung, the filtration flow was maintained at 0.03 L/min for the duration of the experiment. Fluid loss due to ultrafiltration was compensated by adding Plasmalyte solution. The ultrafiltration process helps remove potentially deleterious substances, including activated complement factors, which may contribute to inflammation or lung injury. Additionally, a cytokine adsorber (CytoSorb 300, CytoSorbents Inc. New Jersey, USA) was included in the Cytosorb group. The main differences between our ECMO-based EVLP system and established protocols are summarized in Table 1.

**TABLE 1:** Comparative overview of the ECMO-based EVLP system and conventional EVLP platforms. Adapted from Menander [11].

Parameter	Our protocol (ECMO-based EVLP)	OCS lung system	Toronto Protocol
Perfusion Circuit	Modified ECMO circuit (centrifugal pump, oxygenator, heat exchanger)	Portable OCS circuit (pulsatile flow)	XVIVO circuit (centrifugal pump, oxygenator, heat exchanger)
Priming Solution	Plasmalyte + Albumin + donor blood	Proprietary OCS solution + packed red blood cells (PRBC)	Steen solution
Flow Initiation	Low flow 0.5 L/min → target 0.8–1.0 L/min	Automated gradual ramp-up	10%–40% of predicted cardiac output, adjusted stepwise
Gas mixture	Controlled air flow gas generator (21% O_2_, CO2)	Automated oxygenation and ventilation	8% CO2, 6% O2, 86% N2
Ventilation Mode	Conventional ventilator, lung-protective settings	Integrated ventilator, lung-protective settings	Integratedy ventilator, lung-protective settings
Perfusion Pressure Target	Pulmonary artery pressure <20 mmHg	Pulmonary artery pressure ∼10–20 mmHg	Pulmonary artery pressure <20 mmHg
Anticoagulation	Heparin 50,000 IU (initial)	Heparinized priming solution	Heparin bolus and maintenance doses
Preservation Solution Pre-flush	Plasmalyte	OCS solution	Perfadex or Steen Solution
Assessment Duration	Up to 24 h	Up to 6–8 h	Up to 12 h
Unique Feature	Adaptation of clinical ECMO circuit for EVLP	Portable, fully integrated, transportable	Widely established research and clinical protocol

The trachea was connected to a mechanical ventilator (Zisline MV300, ZetMedical Ltd, USA) via a silicone tube. Pressure-controlled ventilation was applied with a positive end-expiratory pressure (PEEP) of 5 cmH_2_O, an inspiratory pressure of 6 cmH_2_O, a respiratory rate of 10 breaths/min, and an FiO_2_ of 21%. Peak inspiratory pressure (PIP) was limited to 25 cmH_2_O to minimize the risk of barotrauma. A humidification system (VADI VH-1500, VADI Medical Technology Co., Ltd, Taiwan) was employed during mechanical ventilation to maintain adequate airway moisture.

### Randomization

The lungs were randomly allocated into the following groups:

1. Cytosorb group (n = 12), where lungs were perfused with an EVLP circuit containing a Cytosorb cartridge, and Control group (n = 12), where lungs were perfused with an EVLP circuit without a Cytosorb cartridge.
2. The Bronchodilator group (n = 5) consisted of lungs that were nebulized with a combined bronchodilator (Berodual^®^ N, Boehringer Ingelheim, Kiev, Ukraine) every 4 h after the initiation of ventilation. The active components of the bronchodilator included an anticholinergic agent, Ipratropium bromide (20 µg), and a β2-adrenoceptor agonist, Fenoterol hydrobromide (50 µg). The corresponding Control group (n = 5) inhaled the control solvent under the same settings.

### Organ Evaluation

*Functional assessments* were conducted every 2 h, measuring pulmonary artery pressure (PAP), peak airway pressure, dynamic compliance (Cdyn), the ratio of partial pressure of oxygen to the fraction of inspired oxygen (PaO_2_/FiO_2_), and blood gas levels. The lungs were weighed before the initiation of EVLP to establish a baseline and reweighed after the perfusion process to evaluate weight gain.

#### Radiologic Assessment

Following 24 h of perfusion, computed tomography (CT) scans were performed to evaluate lung structure. CT scans were performed using a multi-detector computed tomography (MDCT) system (Siemens Healthineers AG, Germany). Images were independently reviewed by two radiologists blinded to group assignments to minimize bias. Lung injury was assessed using a standardized scoring system [16] defined as follows: 1—absence of consolidation.; 2—Minimal consolidation, involving 1%–25% of the lung; 3—Moderate consolidation, affecting 26%–50% of the lung; 4—Extensive consolidation, covering 51%–75% of the lung; and 5—Near-total consolidation, with 76%–100% of the lung affected. The total score was then calculated and presented.

#### Microscopic Assessment

Lung tissues were collected from the same lower lobe at the end of the EVLP procedure for histological analysis. Tissue samples were fixed in formalin, embedded in paraffin, and sectioned into thick slices. The sections were stained with Hematoxylin and Eosin to assess cellular architecture and lung injury. Microscopic examination was conducted using light microscopy. Histological lung injury was scored based on a standardized injury scale [17]. Lung injury scores were quantified by a blinded investigator. The lung injury score was obtained by summing the scores of five independent variables: intra-alveolar edema, interstitial edema, neutrophil infiltration in the alveolar or interstitial spaces, and the presence of hyaline membranes. Scores for each sample were assigned based on the extent of damage observed: 0 – none, 1 -mild, 2 -moderate, 3 – severe, 4- diffuse.

#### Cytokine Level Monitoring

Cytokine levels, including interleukin-6 (IL-6) and interleukin-8 (IL-8), were analyzed using blood samples drawn from the EVLP circuit at 6-h intervals over the 24-h perfusion period.

### Statistics

All results are expressed as mean ± standard deviation (SD). The Shapiro-Wilk test was used to assess the normality of data distribution. Longitudinal data trends between groups were analyzed using a two-way analysis of variance (ANOVA). Comparisons of continuous outcomes between groups at specific time points were conducted using Mann-Whitney U tests. A p-value of <0.05 was considered statistically significant. All analyses were performed using GraphPad Prism version 10.4 software.

## Results

The primary aim of this study was to establish and evaluate an innovative, alternative EVLP system based on an ECMO circuit. Our results demonstrate that this ECMO-based EVLP setup enabled stable perfusion and ventilation of donor lungs over 24 h, maintaining physiological parameters and functional stability comparable to established EVLP protocols.

### Functional Assessment

**FIGURE 1:**
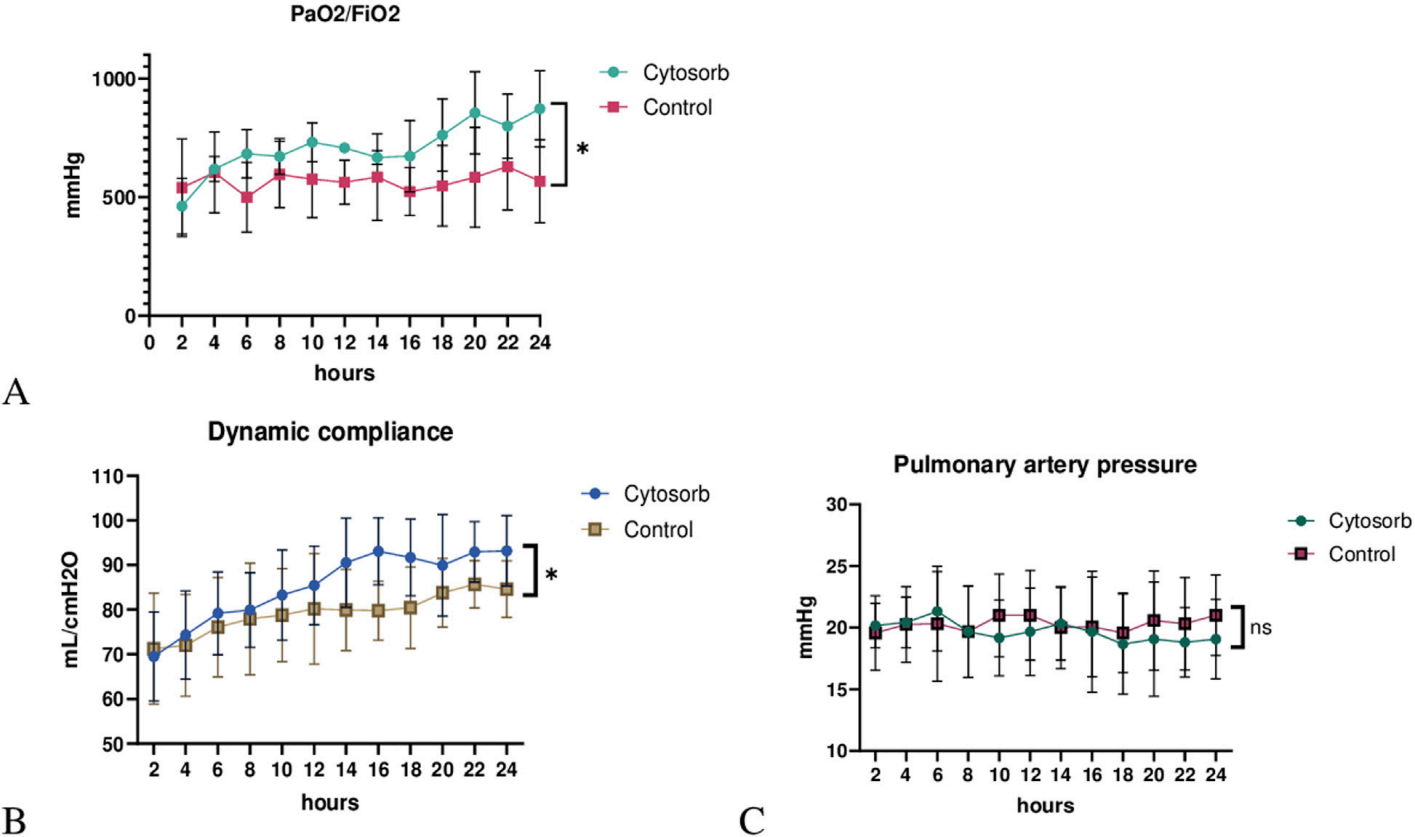
Lung physiology during *ex vivo* lung perfusion. **(A)** The PaO_2_/FiO_2_ ratio was significantly higher, and **(B)** dynamic compliance was markedly improved in the Cytosorb group compared to the Control group. **(C)** Pulmonary artery pressure (PAP) showed no significant difference between groups. Results are shown as mean ± standard deviation, analyzed using 2-way ANOVA (*p < 0.05).

Pulmonary artery pressure (PAP), peak airway pressure, dynamic compliance (Cdyn), and the ratio of partial pressure of oxygen to the fraction of inspired oxygen (PaO_2_/FiO_2_) were recorded at 2-h intervals throughout the EVLP period. Gas exchange efficiency, as measured by the PaO_2_/FiO_2_ ratio, remained consistently above 300 in both groups over the 24-h perfusion period, indicating lung viability. However, PaO_2_/FiO_2_ ratio was significantly higher in the Cytosorb group than control (p < 0.05; Figure 1A). Moreover, lung mechanics, as determined by dynamic compliance, was significantly better in the Cytosorb group (p < 0.05; Figure 1B). Pulmonary artery pressure demonstrated similar trends over time and no difference was observed between groups (P = 0.9; Figure 1C).

**FIGURE 2:**
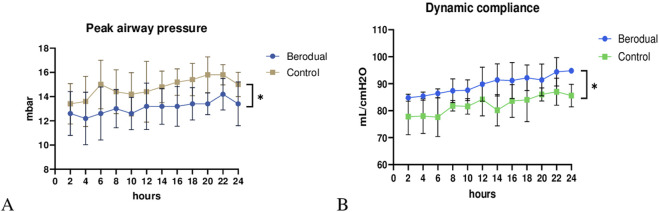
Functional assesment during *ex vivo* lung perfusion. Berodual group had significantly lower peak airway pressure **(A)** and higher dynamic compliance **(B)** than the Control group (*p < 0.05).

Peak airway pressure (Figure 2A) was significantly lower in the Bronchodilator group compared to the Control group (p < 0.05). Dynamic compliance (Figure 2B) significantly improved in the Bronchodilator group (94.8 ± 0.8 mL/cmH_2_O) compared to the Control group (86.6 ± 3.2 mL/cmH_2_O, p < 0.05) by the end of the 24-h perfusion period. Notably, dynamic compliance in the Bronchodilator group showed a steady upward trend, suggesting progressive improvement in lung mechanics.

### Radiological Assessment

**FIGURE 3:**
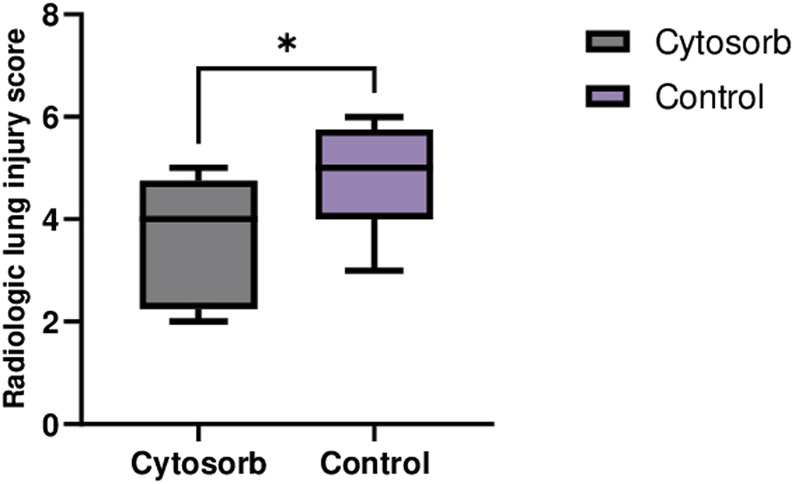
Computed Tomography of Lungs on EVLP System. CT scan images at the end of perfusion revealed significantly lower lung injury in the Cytosorb group compared to the Control group. The data are presented as the mean ± SD, and statistical significance was assessed using the Mann–Whitney U-test (*p < 0.05).

The CT images in the Cytosorb group showed better-preserved lung tissue and less edema, while the control group exhibited more areas of consolidation (Figure 3). The differences in lung injury between the two groups were statistically significant (Cytosorb: 3.5 ± 1.1 vs. Control: 4.6 ± 1.0; p < 0.05).

### Histological Analysis

**FIGURE 4:**
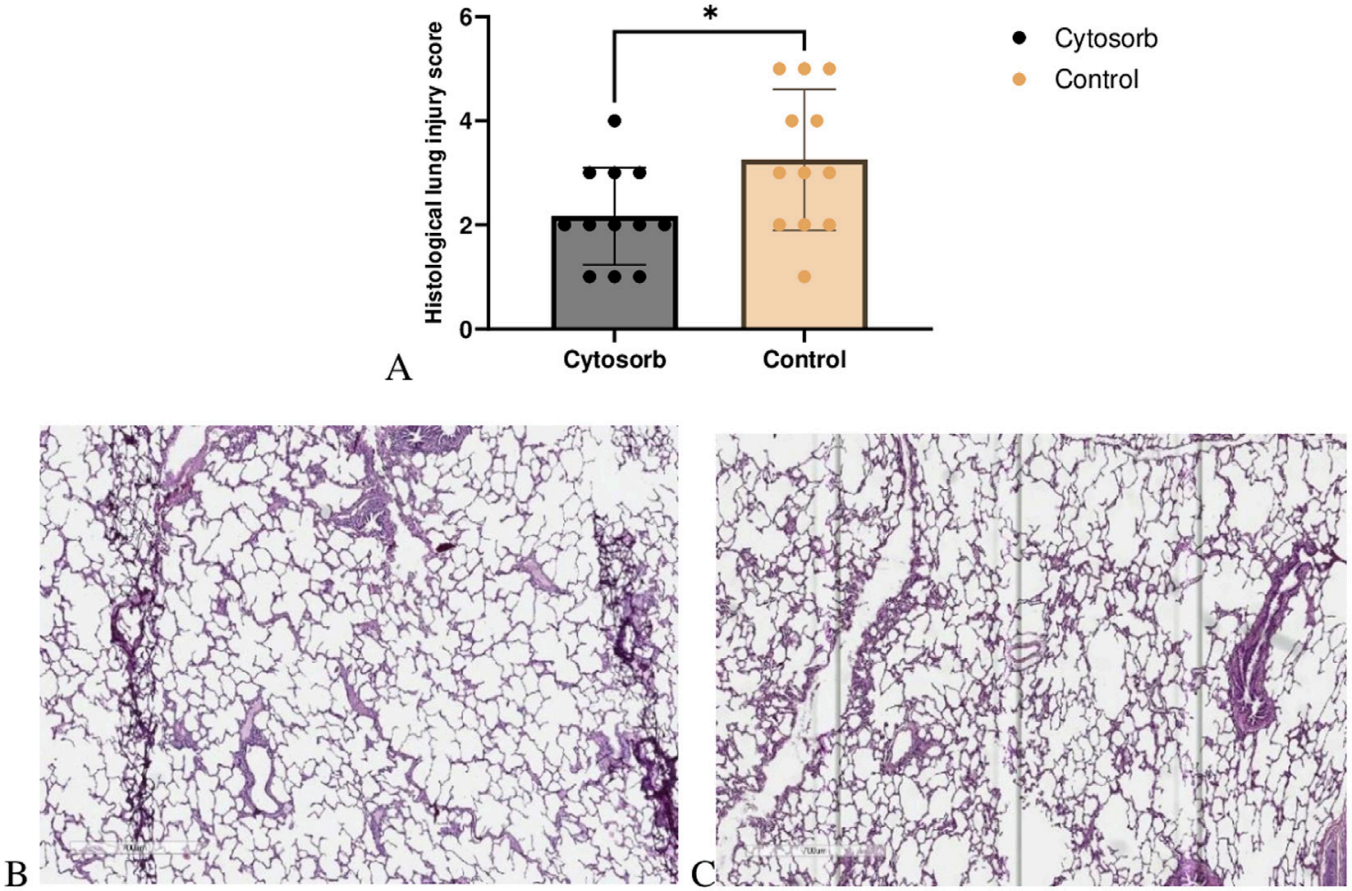
Microscopic assessment of lung injury. **(A)** Histological lung injury scores. The Cytosorb group demonstrated a significant reduction in lung injury scores compared to the control group. Data are expressed as means ± SD (Mann–Whitney U‐test, *p < 0.05). Lung tissues were stained with H&E, and slides from the Cytosorb group **(B)** and the control group **(C)** were examined under light microscopy at an original magnification of ×700.

Histological lung injury scores were significantly lower in the Cytosorb group compared to the control group (Cytosorb: 2.1 ± 0.9 vs. Control: 3.2 ± 1.3; p < 0.05). Microscopic examination of the Cytosorb group revealed well-preserved alveolar architecture with minimal disruption to the alveolar-capillary barrier. In contrast, lung injury in the control group was characterized by more extensive interstitial edema at the end of EVLP. Notably, no evidence of alveolar or interstitial hemorrhage was observed in any of the samples (Figure 4).

### Weight Gain

**FIGURE 5:**
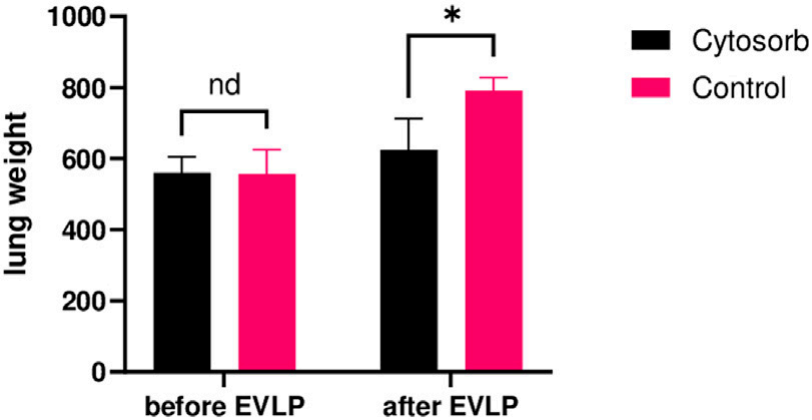
Lung weights after EVLP. The Cytosorb group gained less weight than the Control group after 24 h of *ex-vivo* perfusion. Multiple Mann-Whitney tests were used. “nd” indicates no significant difference. *p < 0.05.

Lung weight was measured before and after the 24-h EVLP period (Figure 5). Baseline lung weight did not differ significantly between the 2 groups (Cytosorb 559 ± 45g, Control 556 ± 69g; p = 0.89). By the end of the run, the Cytosorb group exhibited the least weight gain than the Control group (11.5% and 42.2 respectively, p < 0.01).

### Cytokine Analysis

**FIGURE 6:**
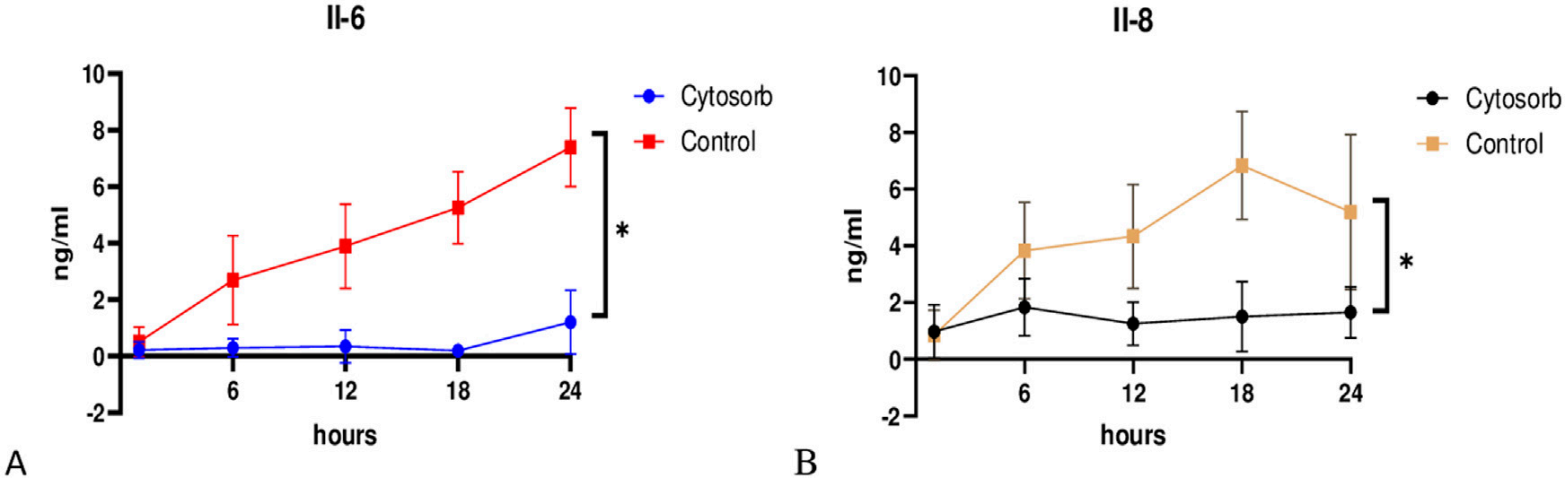
Cytokine profile during EVLP. Cytosorb group significantly reduced interleukin IL-6 **(A)** and IL-8 **(B)** levels compared to the control group throughout the perfusion. Data are presented as mean ± standard deviation. Two-way analysis of variance (ANOVA) with *p < 0.05 was used for comparisons.

Cytokine analysis revealed that levels of IL-6 and IL-8 were significantly lower in the Cytosorb group compared to the control group during EVLP, indicating a potential reduction in inflammatory response (Figure 6). These findings provide insight into the modulation of inflammation and immune response during prolonged perfusion.

## Discussion

The use of EVLP has significantly advanced the field of lung transplantation by providing a platform to assess and optimize donor lungs before transplantation. Functional parameters such as oxygenation capacity, pulmonary vascular resistance, and lung compliance during EVLP have been shown to correlate with post-transplant graft function. Studies have demonstrated that lungs meeting EVLP criteria for transplantation exhibit favorable early post-transplant outcomes, including lower rates of primary graft dysfunction and improved short-term survival [18].

In this study, we explored the use of an ECMO-based system as an alternative platform for EVLP. Unlike conventional EVLP circuits, which rely on custom-built perfusion setups and specialized equipment, the use of an ECMO system offers several potential advantages for donor lung preservation, including simplified circuit assembly, clinical familiarity, widespread availability, and integration into transplant logistics.

While the effects of adjunctive therapies such as Cytosorb adsorption and bronchodilator administration have been reported in the literature, the application of these interventions in an ECMO-based *ex vivo* perfusion model represents a novel approach. Our findings suggest that this system could serve as a reliable alternative to traditional EVLP machines and may contribute to expanding the donor lung pool by improving preservation strategies.

Cytokines, signaling proteins released during IRI, intensify inflammation by attracting leukocytes to the injury site [19]. Elevated levels of cytokines, particularly interleukin-6 (IL-6) and interleukin-8 (IL-8), in bronchoalveolar lavage fluid immediately after lung transplantation, are associated with poor short-term and long-term graft outcomes [20]. During *ex vivo* lung perfusion (EVLP), increased IL-8 levels have been correlated with the development of PGD [21]. EVLP offers a valuable platform for reducing ischemia-reperfusion injury by allowing therapeutic interventions during lung evaluation and treatment [22].

The findings from this study highlight the potential of combining advanced technologies, such as the Cytosorb cartridge to improve outcomes during EVLP. The Cytosorb group exhibited superior lung mechanics, as evidenced by a significantly higher PaO_2_/FiO_2_ ratio and improved dynamic compliance. These outcomes align with previous research with acellular perfusate indicating the efficacy of cytokine filtration in mitigating inflammation and reducing lung injury during EVLP [23]. Clinically, cytokine filtration has demonstrated the potential to improve oxygenation and reduce the incidence and severity of PGD following lung transplantation [24, 25].

Another promising approach to attenuate IRI during EVLP involves the use of β2-adrenoreceptor agonist inhalation [26]. β2-adrenergic receptors (β2-AR) are densely distributed throughout the lungs and play a critical role in regulating various cellular functions. In airway smooth muscle, β2-AR activation induces bronchodilation by increasing cyclic adenosine monophosphate (cAMP) levels. In the alveolar epithelium, β2-AR stimulation enhances ion transport, aiding in the clearance of excess alveolar fluid. Furthermore, activation in type II alveolar cells promotes surfactant production, which is essential for maintaining lung compliance [27]. In endothelial cells, β2-AR activation regulates nitric oxide-dependent vasodilation and reduces vascular permeability [28]. Additionally, β2-AR stimulation in mast cells suppresses airway inflammation by inhibiting histamine release [29].

Anticholinergic agents, which inhibit muscarinic receptors, also contribute to pulmonary protection. In addition to bronchodilation, these agents have demonstrated protective effects in experimental models of lung IRI. They mitigate oxidative stress [17], reduce neutrophil migration to sites of inflammation [16, 30], and downregulate matrix metalloproteinase-9 (MMP-9) activity [16], suggesting potential benefits beyond their primary role in bronchodilation.

Reconditioning lung grafts with nebulized β2-adrenomimetics during EVLP has shown promising results. Studies in canine models indicate that inhalation of β2-AR agonists during EVLP significantly reduces lung injury [26] and improves post-transplant graft function [31]. However, the combined use of β2-adrenomimetics and anticholinergic agents during extended EVLP remains unexplored.

Our study demonstrates that whole blood based perfusate with continuous ultrafiltration for functioning lung in machine perfusion can be straightforward and efficient for at least 24 h. Additional cytokine removal had led to significant improvement of organ quality (morphological and functional) over 24 h of EVLP. By the end of EVLP, perfusate sodium concentrations were in physiological levels in contrast to the study of Iskander et al [23].

During this study we did not observe neither metabolite accumulation, nor electrolyte imbalance in perfusate. There are critical thresholds of these parameters as the primary driver of the deterioration in compliance over EVLP have been established in previous works [9]. All groups demonstrated stable electrolyte, metabolic and immunological homeostasis. The latter effect was more pronounced in immunomodulation (Cyto-Sorb) group in terms of weight gain, lung injury scores by CT and microscopic assessment, lung physiology.

Overall, our results strongly support significant morphological and functional benefit of continuous ultrafiltration in combination with cytokine removal and bronchodilatation during 24 h EVLP. These effective tools enable future therapeutic possibilities for extended criteria donation, long term organ transportation, and even organ banking.

### Limitations

One of the limitations of our study was the 24 h limited EVLP. Further studies looking at metabolism, immunomodulation are needed to extend perfusion significantly beyond 24 h. Our current study did not test combining bronchodilators with Cytosorb, but we recognize its potential clinical significance. Future research should investigate the synergistic effects of this combination during EVLP to determine whether it enhances lung compliance, reduces inflammation, and ultimately improves post-transplant graft function and success rates. Another limitation was absence of LTx after EVLP. Although our study does not include post-transplant follow-up, the functional viability observed during EVLP suggests a strong potential for successful graft performance *in vivo*. Future research incorporating LTx and post-transplant follow-up is needed to validate these findings further and enhance the clinical relevance of EVLP as a reliable predictor of post-transplant outcomes. Our group is constantly progressing on this issue.

## Data Availability

The original contributions presented in the study are included in the article/supplementary material, further inquiries can be directed to the corresponding author.
